# ADHD and Intellectual Disability: using ADHD medication

**DOI:** 10.1192/j.eurpsy.2022.252

**Published:** 2022-09-01

**Authors:** K. Courtenay, B. Perera

**Affiliations:** 1 Barnet Enfield and Haringey Mental Health NHS Trust, London, UK, Psychiatry - Nlfs, London, United Kingdom; 2 Royal College of Psychiatrists, Faculty Of Psychiatry Of Intellectual Disability, BB, United Kingdom; 3 Barnet Enfield and Haringey Mental Health NHS Trust, London, UK, Haringey Ldp, London, United Kingdom

**Keywords:** challenging behaviour, intellectual disability, adhd, medication

## Abstract

**Introduction:**

Mental disorders and ADHD in people with ID are higher than in the general population.Clinicians may be reluctant to diagnose ADHD in people with ID. They could be denied effective treatment.

**Objectives:**

The purpose of the study was to ascertain antipsychotic use in people with ID before and after the a diagnosis of ADHD.

**Methods:**

A casenote review in an ID service for aduls with ADHD. Data collected on psychotropic use before and after the diagnosis.

**Results:**

Forty-eight aduls with ADHD-ID were identified. 38(79%) were male and 10(21%) were female. 19 to 58 years of age. Four (8%) had mild ID; 44 (92%) had moderate to severe ID. 27(56%) had anxiety, mood disorders or psychosis. 21(44%) had ADHD only. Challenging behaviour was reported in 24 (50%) of cases. Thirty-three (68%) used psychotropic medication prior to the diagnosis of ADHD and after the diagnosis. Post-diagnosis, 20(60%) continued to use antipsychotic medication indicating the elimiation of antipsychotic use in 13(40%) of people. The level of medication use remained the same in spite of the reduction of antipsychotic medication. The diagnoses of challenging behaviour was not affected by the reduction in antipsychotic medication and the increase in ADHD medication use.

**Conclusions:**

The use of antipsychotic medication in people with intellectual disaibilities and ADHD is high. ADHD should be considered when people present wtih challenging behaviour. ADHD medication can be effective in treating ADHD-ID and can lead to a significant reduction in the use of antipsychotic medication.

**Disclosure:**

No significant relationships.

